# AI-2E Family Transporter Protein in *Lactobacillus acidophilus* Exhibits AI-2 Exporter Activity and Relate With Intestinal Juice Resistance of the Strain

**DOI:** 10.3389/fmicb.2022.908145

**Published:** 2022-05-12

**Authors:** Xiefei Li, Xiankang Fan, Zihang Shi, Jue Xu, Yingying Cao, Tao Zhang, Daodong Pan

**Affiliations:** ^1^State Key Laboratory for Managing Biotic and Chemical Threats to the Quality and Safety of Agro-Products, Ningbo University, Ningbo, China; ^2^Key Laboratory of Animal Protein Deep Processing Technology of Zhejiang, Ningbo University, Ningbo, China

**Keywords:** AI-2E family transporter, *Lactobacillus acidophilus*, overexpression, intestinal juice, real-time quantitative PCR, AI-2

## Abstract

The function of the autoinducer-2 exporters (AI-2E) family transporter protein of *Lactobacillus acidophilus* is still unclear. The phylogenetic analysis was used to analyze the relationship between the AI-2E protein of the *L. acidophilus* CICC 6074 strain and other AI-2E family members. *Escherichia coli* KNabc strain was used to verify whether the protein has Na^+^ (Li^+^)/H^+^ antiporter activity. The AI-2E protein overexpression strain was constructed by using the pMG36e expression vector, and the overexpression efficiency was determined by real-time quantitative PCR. The vitality and AI-2 activity of *L. acidophilus* CICC 6074 strains were determined. The results showed that the AI-2E protein of *Lactobacillus* formed a single branch on the phylogenetic tree and was closer to the AI-2E family members whose function was AI-2 exporter group I. The expression of AI-2E protein in the *E. coli* KNabc strain did not recover the resistance of the bacteria to the saline environment. Overexpression of AI-2E protein in *L. acidophilus* CICC 6074 could promote the AI-2 secretion of *L. acidophilus* CICC 6074 strain and enhance their survival ability in intestinal juice.

## Introduction

The well-known probiotic effect of probiotics has made it widely accepted by consumers and has formed a massive market on a global scale (Nguyen et al., 2020). The health benefits of lactic acid bacteria (LAB) and their positive effects on food have led to their addition to various food products as a probiotic. As a representative of probiotics, the probiotic effects of LAB in maintaining the balance of the gastrointestinal tract, modulating the immune system, reducing lactose intolerance, and preventing diarrhea have been widely confirmed (Michael et al., 2001; Jin et al., 2010; Kostelac et al., 2021). *Lactobacillus* can only exert positive probiotic effects on the human body when sufficient amounts of live bacteria are ingested and safely pass through the digestive tract to reach the intestines and colonize. Studies have found that the viability of probiotic bacteria should be at least 10^7^ CFU/g when consuming probiotic products (Ranadheera et al., 2010). The vitality of probiotics is the key factor to play the probiotic effect, so the research to improve the vitality of probiotics is of great significance.

Factors affecting the viability of probiotics have been extensively studied, and researchers have thus proposed several methods to enhance the viability of LAB in the gastrointestinal tract. Among the common methods is the development of different probiotic food carriers, the screening of probiotic strains with good resistance, the optimization of probiotic production and storage conditions, and microencapsulation of bacterial cells. Compared to fermented milk, cheese has the advantages of higher pH, higher fat content, lower oxygen content, and a dense woven matrix, which is thought more effective in delivering probiotics into the human gut (Karimi et al., 2011). Sakamoto et al. (2001) screened 203 strains of *Lactobacilli* to select strains that exhibited high levels of resistance to gastric acidity, the *Lactobacillus gasseri* OLL2716 has been selected as the best strain, and a yogurt product that inhibits *Helicobacter pylori* was developed from this strain. Microencapsulation is a relatively effective method to improve the survival rate of LAB, which has been studied by many researchers (Damodharan et al., 2017; Ghibaudo et al., 2017; Chen et al., 2020).

Another way that could improve adaptation to the gastrointestinal fluid environment of LAB is to focus on the bacteria themselves and strain improvement. Several researchers have identified and confirmed that some genes or genetic factors are associated with the resistance of LAB (Azcarate-Peril et al., 2004; Fiocco et al., 2007; Bove et al., 2012). Patnaik et al. (2002) used the genomic recombination method to improve the acid tolerance of an industrial strain of *Lactobacillus* and obtained a mutant strain that grew on liquid and solid media at a much lower pH than the wild-type strain. Pfeiler et al. reported that deletion mutations in proteins such as transporter and *HPK* resulted in the loss of bile tolerance in *Lactobacillus acidophilus* NCFM strains, and mutations in an oxidoreductase and hypothetical protein genes resulted in a significant increase in bile tolerance in the strains (Pfeiler et al., 2007; Pfeiler and Klaenhammer, 2009). Goh et al. (2009) found that the Apf-like proteins may contribute to the survival of *L. acidophilus* during transit through the digestive tract and, potentially, participate in the interactions with the host intestinal mucosa.

Transmembrane transport is involved in nutrient uptake, metabolite excretion, energy production, and conversion in bacterial cells and is essential for bacteria to adapt to their environment (Lorca et al., 2007). The autoinducer-2 exporters (AI-2E) family homologs are widely distributed in various bacteria and archaea and are a common transporter protein encoded by genes on the bacterial chromosome (Rettner and Saier Jr, 2010). All members of AI-2E protein in the AI-2E family transporter have a uniform 8 transmembrane helices (TMHs), with the second 4 TMHs being more conserved than the first 4 TMHs. The functions of AI-2E have been identified mainly for two main categories, exporting autoinducer-2 (AI-2) molecules and acting as Na^+^ (Li^+^)/H^+^ antiporter (Rettner and Saier Jr, 2010; Dong et al., 2017). These two functions of AI-2E are thought to be related to microbial lifestyle and environmental adaptability. Wang et al. (2020) used proteomics to find that AI-2E proteins were closely associated with the survival of *Acinetobacter johnsonii* and *Pseudomonas fluorescens* in the environment (Wang and Xie, 2020). Shao et al. (2018) found that the AI-2E protein with Na+/H+ antiporters activity was associated with *Halobacillus andaensis* resistance to high saline-alkaline stress (Shao et al., 2018).

The AI-2E proteins are present in almost all LAB strains, and the function and mechanism of action of AI-2E protein in LAB are rarely mentioned. Whether it is related to the adaptation of LAB to the environment has also been little studied. Lorca et al. (2007) identified the AI-2E protein as serving to export AI-2 in 11 bacterial species containing *L. casei* but not *L. acidophilus*. In one of our studies, we observed a significant upregulation of the LBA_RS00210 gene in *L. acidophilus* that had been exposed to intestinal juice (data unpublished). The effect of AI-2E protein on the ability of LAB to survive in the intestine has not yet been studied. Therefore, we verified the function of the AI-2E protein in *L. acidophilus* and found that this protein affects the viability of the bacteria cells in the intestinal juice.

## Materials and Methods

### Bacteria Strains and Growth Conditions

The *E. coli* KNabc strain was grown in the KCl-modified Luria-Bertani (LBK) medium (Dong et al., 2017), and *E. coli* DH5α was cultured in the Luria–Bertani (LB) broth. *E. coli* competent cells were prepared with the Competent Cell Preparation Kit [Takara Biomedical Technology (Beijing) Co., Ltd., China], and the transformants were selected by ampicillin with a concentration of 100 μg/ml. Salt and alkaline pH tolerance of *E. coli* KNabc strain were tested as described in the studies of Shao et al. (2018). The *L. acidophilus* CICC 6074 strain was grown in the Man, Rogosa and Sharpe (MRS) medium. The mutant strain overexpressing the AI-2E protein was selectively grown in the MRS medium containing 5 μg/ml erythromycin. Intestinal juice tolerance experiments of *Lactobacillus* cells were performed as described in the studies of Maragkoudakis et al. (2006) and Ranadheera et al. (2014).

### Bioinformatic Analyses

MEGA (Version 11.0.10) was used to construct the phylogenetic tree to analyze the functional and taxonomic information of the AI-2E protein of *Lactobacillus* (a neighbor-joining phylogenetic tree with a bootstrap analysis 1,000 replications). The AI-2E family transporter protein sequences of *Lactobacillus* were downloaded from the Identical Protein Groups (IPG) of NCBI, and the sequences of proteins belonging to the AI-2E family (TC #9.B.22) were downloaded from the transporter classification database (TCDB). The homolog protein of the AI-2E family transporter from *L. acidophilus* CICC 6074 was obtained by performed blastp and downloaded from NCBI. Topological analysis was carried out on SMART (http://smart.embl-heidelberg.de/), PredictProtein (https://predictprotein.org/), and Geneious. Weblogo was created with Geneious.

### Construction of Recombinant Plasmid

To be able to express AI-2E protein in *E. coli* cells, we used the pUC19 plasmid to ligate the *AI-2E* gene fragment containing the promoter sequence. To express the AI-2E protein with 6×His tag in *E. coli* cells and to take the strain ampicillin-resistant, we used the pET22b plasmid and replaced the pelB tag on the vector with the 6×His tag. And to overexpress AI-2E protein in cells of *L. acidophilus* CICC 6074, the pMG36e plasmid was used by us. This vector is known as a food-grade LAB expression vector with the advantages of simple structure, clean expression product, strong promoter, and no need to be induced. Many researchers have used this vector when expressing proteins in LAB strains. Geneious software was used to locate the AI-2E gene on the genome of *L. acidophilus*, and the TSSG program (http://www.softberry.com/berry.phtml?topic=tssg&group=programs&subgroup=promoter) and BDGP: Neural Network Promoter Prediction website (https://www.fruitfly.org/seq_tools/promoter.html) were used for promoter prediction. CE Design V1.04 software (Vazyme Biotech Co., Ltd., China) was used to design-specific primers for amplifying the AI-2E gene. The target gene was amplified using the 2 × Phanta Max Master Mix (Vazyme Biotech Co., Ltd., China), and the amplification products were recovered and purified using the Ultra-Sep Gel Extraction Kit (Omega Biotek Inc., USA). The plasmids were extracted using the HP Plasmid DNA Midi Kit (Omega Biotek Inc., USA), and the Takara QuickCut restriction enzyme [Takara Biomedical Technology (Beijing) Co., Ltd., China] was used to digest the three vectors separately. The prepared target gene fragment and the linearized vector fragment were ligated using the ClonExpress^®^ Ultra One Step Cloning Kit (Vazyme Biotech Co., Ltd., China). The constructed plasmids were sequenced to verify the ligation and ensure that the target gene was not mutated.

### Transformation and Electrotransformation

The *E. coli* KNabc is a strain lacking the three major Na^+^ (Li^+^)/H^+^ antiporters (Δ*nhaA*, Δ*nhaB*, and Δ*chaA*). The Na^+^ (Li^+^)/H^+^ antiporter activity of the *AI-2E* genes can be verified by transferring and expressing these genes in *E. coli* KNabc strain to recover the resistance of strain cells to the saline environment (Majernik et al., 2001; Dong et al., 2017). Therefore, we used *E. coli* KNabc strain to verify the Na^+^ (Li^+^)/H^+^ antiporter activity of AI-2E protein of *L. acidophilus*, and *L. acidophilus* CICC 6074 strain was used to overexpress AI-2E protein. For transformation, 100 μl of *E. coli* KNabc competent cells were melted on ice, 10 μl of recombinant plasmid was added to the competent cells. The mixture of competent cells with the recombinant plasmid was ice bathed for 30 min and then heat shock in hot water at 42°C for 45 s. Preparation of *L. acidophilus* competent cells and electroporation were carried out with some modifications of the method from Palomino et al. (2010). The MicroPulser Electroporator (Bio-Rad Laboratories, Inc., USA) was used to perform the electroporation with the parameters set to 12.5 kV/cm, 4 ms. We mixed 50 μl of *L. acidophilus* competent cells with 5 μl of the recombinant plasmid. The mixture was added to pre-cooled 0.2-cm electrode gap cuvettes and then performed electroporation according to the set parameters.

### Na^+^ (Li^+^)/H^+^ Antiporter Assay

The *E. coli* KNabc wild-type strain and the recombinant strains (*E. coli* KNabc/pUC19, *E. coli* KNabc/pET22b, *E. coli* KNabc/pUC19-AI-2E, and *E. coli* KNabc/pET22b-AI-2E) were streak cultured on fresh and 100 μg/ml ampicillin-added LBK agar plates, respectively. Single colonies were picked for the expansion. To determine the resistance of the *E. coli* KNabc strain to alkaline pH, Na^+^ and Li^+^; the *E. coli* KNabc strain and recombinant strains were activated, followed by 1% (v/v) subculture in the LBK medium at different pH (7.0, 7.5, 8.0, and 8.5) and the LBK medium at pH 7.0 but with different concentrations of NaCl (0.2, 0.3, and 0.4 M) or LiCl (5, 10, 25, and 30 mM). After incubation at 37°C for 24 h, the optical density (OD_600_) values of the bacterial culture were measured by Infinite M200 PRO Multimode Microplate Reader (Tecan Group Ltd., Switzerland). Three replicates were done for each sample.

### Real-Time Quantitative PCR

After being cultured at 37°C for 18 h, the total RNA of the *L. acidophilus* CICC 6074 wild-type strain and the recombinant strains (*L. acidophilus* CICC 6074/pMG36e and *L. acidophilus* CICC 6074/pMG36e-AI-2E) was extracted using E.Z.N.A.^®^ Bacterial RNA Kit (Omega Biotek Inc., USA). The concentration of RNA was measured by NanoDrop ND-2000 Spectrophotometer (Thermo Fisher Scientific Inc. USA), and RNA integrity was observed by agarose gel electrophoresis. Genomic DNA removal and cDNA synthesis were accomplished using HiScript^®^ III RT SuperMix for qPCR (+gDNA wiper) (Vazyme Biotech Co., Ltd, China). Real-time quantitative PCR (RT-qPCR) was performed using Taq Pro Universal SYBR qPCR Master Mix (Vazyme Biotech Co., Ltd, China) on LightCycler^®^ 96 Instrument (Roche Molecular Systems, Inc., Switzerland). Primers used in RT-qPCR were designed using Primer premier 6, DNA polymerase III subunit delta gene was used as reference gene. The Livak (2^−Δ*ΔCT*^) method was used to calculate the relative expression of the *AI-2E* gene.

### AI-2 Detection

The detection of AI-2 is based on the method of Liu et al. (2018) with some modifications. Activated *L. acidophilus* CICC 6074 wild-type strain and recombinant strains (*L. acidophilus* CICC 6074/pMG36e and *L. acidophilus* CICC 6074/pMG36e-AI-2E) were added to 12% (w/w) skimmed milk medium without erythromycin. The *L. acidophilus* cells in cultures at 2, 4, 6, 8, 10, 12, 14, 16, 18, 20, 22, and 24 h were removed by centrifugation at 12,000 × *g* for 10 min at 4°C. The supernatant pH was adjusted to 7.0 and filtered by 0.22 μm Millex-GP Syringe Filter Unit (Merck KGaA, Germany). The obtained supernatant was added to *Vibrio harveyi* BB170 and the AB medium mixture in a ratio of 1:10 and incubated at 28°C for 5 h. Added 100 μl of culture solution to a 96-well white plate and determined luminescence values using the Infinite M200 PRO Multimode Microplate Reader (Tecan Group Ltd., Switzerland).

### Stress Resistance Assay

To assess the effect of overexpressing AI-2E protein in *L. acidophilus* on the viability of cells in intestinal juice, we tested the survival of different strains during intestinal fluid tolerance. Injected 2% (v/v) activated *L. acidophilus* CICC 6074 wild-type strain and recombinant strains (*L. acidophilus* CICC 6074/pMG36e and *L. acidophilus* CICC 6074/pMG36e-AI-2E) into fresh MRS medium, incubated at 37°C for 18 h to logarithmic phase. The cells were centrifuged at 5,000 × *g* for 5 min at 4°C, washed three times with sterilized phosphate-buffered saline (PBS), and resuspended with the small intestine solution. The cultures were taken out at 0, 2, 4, and 6 h to count the number of viable bacteria by plate counting method. The survival rates were calculated as follows: the logarithm of survival cell number/the logarithm of initial cell number × 100%. The initial cell number was survival cell at 0 h.

### Statistical Analysis

Statistical analysis was performed using IBM SPSS Statistics 26, and differences were considered statistically significant when the *p*-value was < 0.05. One-way analysis of variance (ANOVA) was used for data analysis. Results are presented as the mean of three independent experiments.

## Results

### Phylogenetic Analysis

**Figure 1:**
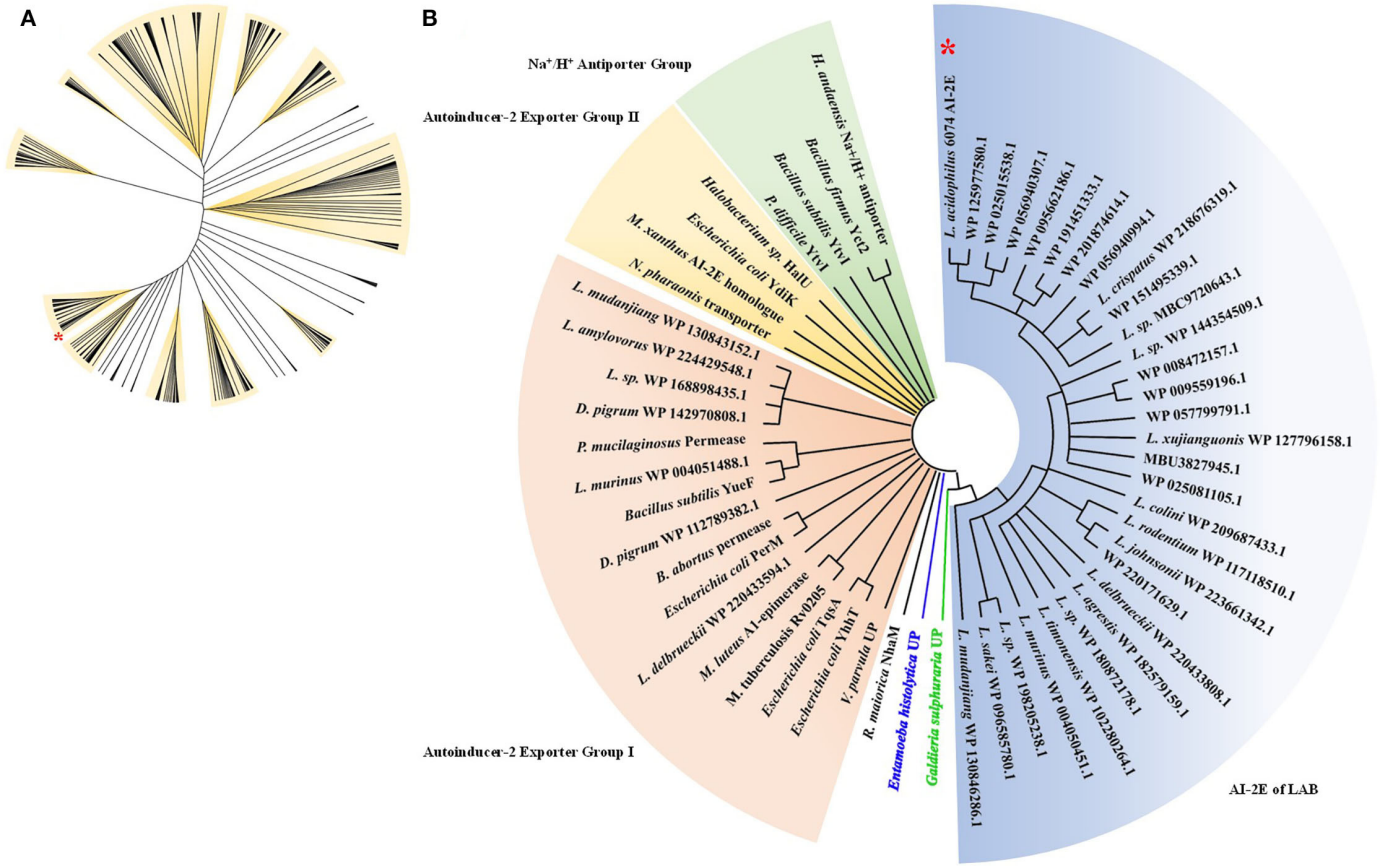
Phylogenetic analysis of AI-2E family transporter protein of *Lactobacillus acidophilus* CICC 6074. Phylogenetic relationship between AI-2E proteins of *Lactobacillus* **(A)**, 530 AI-2E protein sequences of *Lactobacillus* were downloaded from IPG of NCBI, the AI-2E protein of *L*. *acidophilus* is marked with a red star. Phylogenetic relationship between AI-2E protein of *L. acidophilus* CICC 6074 and AI-2E family members **(B)**, phylogenetic analysis using the AI-2E protein of *L*. *acidophilus* with its homologous proteins, and the AI-2E protein of *L. acidophilus* is marked with a red star.

To analyze the relationship between the AI-2E family transporter protein of *Lactobacillus*, 530 sequences were selected and downloaded for phylogenetic analysis, and phylogenetic trees were constructed by the neighbor-joining method. The result of the analysis is shown in Figure 1A. We can see that the sequences are divided into 11 branches and some minor branches, and the branch where the AI-2E family transporter protein of *L. acidophilus* CICC 6074 is marked with a red asterisk. There are 52 sequences in the branch where *L. acidophilus* CICC 6074 is, and these sequences mainly belong to four species (*L*. *acidophilus, L*. *helveticus, L*. *gigeriorum*, and *Lactobacillus* sp. A27). These results indicate that there is also some variability in AI-2E proteins of *Lactobacillus*, which also suggests that AI-2E protein of different LAB may have different functions. We constructed a neighbor-joining tree of representative AI-2E protein sequences in *Lactobacillus* with other AI-2E protein sequences from TCDB, results shown in Figure 1B. It shows that the functions of AI-2E family transporter proteins are divided into three categories, AI-2 exporter group I, AI-2 exporter group II, and Na^+^ (Li^+^)/H^+^ antiporter group. Most of the AI-2E proteins from *Lactobacillus* on the phylogenetic tree are assigned to one branch and are closest to group I. There are also 6 AI-2E proteins from *Lactobacillus* assigned to AI-2 exporter group I. These results suggest that the functions of AI-2E protein from *L. acidophilus* are most likely as AI-2 exporter, but further evidence is needed to prove this.

### Structural Domain Prediction of AI-2E Protein of *L. acidophilus*

**Figure 2:**
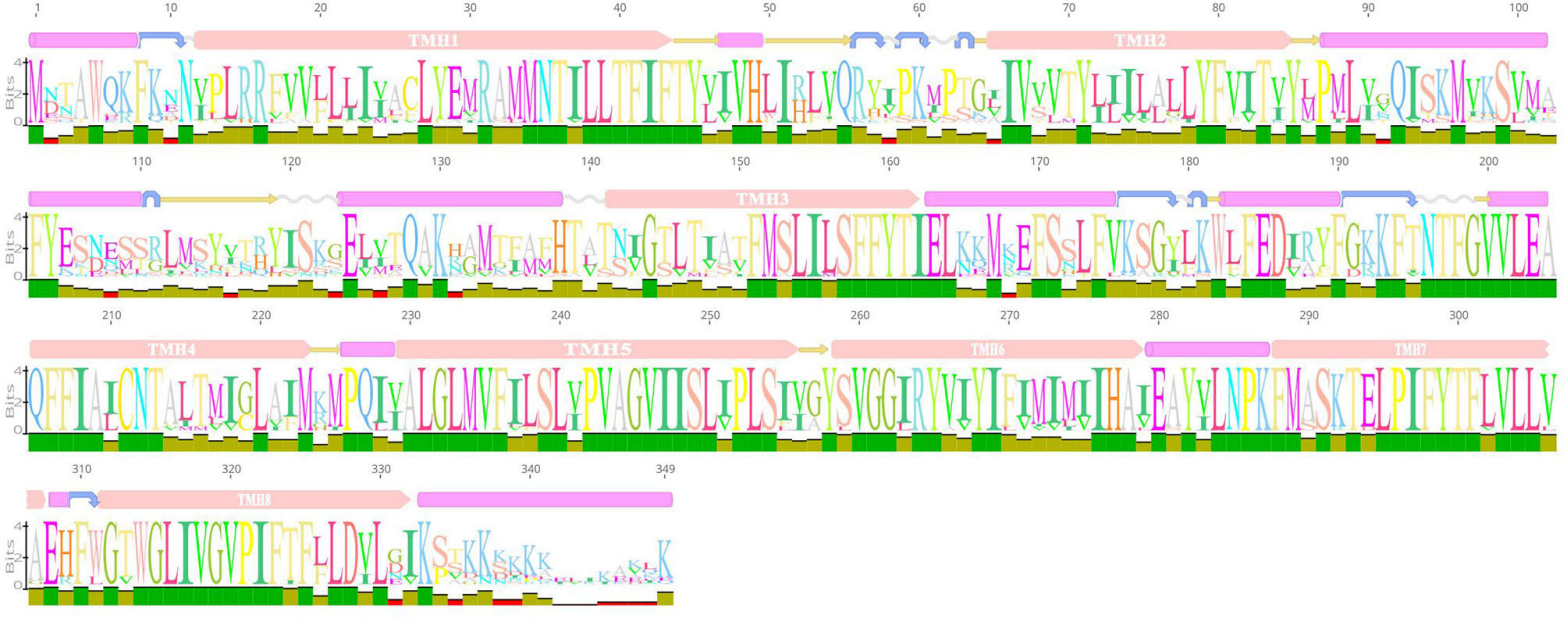
Weblogo of AI-2E protein of *Lactobacillus acidophilus* CICC 6074 and its homologs. The first layer shows the predicted secondary structure and functional domains of AI-2E protein. The pink bars represent an α-helix, the blue arrows represent the corner, the yellow arrows represent the β-fold, and the pink bars marked with transmembrane helix (TMH) are the predicted transmembrane helices. The letters of different sizes in the second layer represent the size of similarity of homologous sequences at that locus, larger letter means higher similarity. The color block in the third layer represents the conservativeness of the sequence and the green color represents that the region is conserved.

We predicted the secondary structure and domains of the AI-2E protein from *L. acidophilus* CICC 6074, and the results are shown in Figure 2. The results show that this protein sequence contains an AI-2E transport structural domain and has eight transmembrane regions. The AI-2E protein sequence of *L. acidophilus* was multiple alignments with 14 homologous proteins. A weblog plot was created to look at the conserved regions of the protein with the structure. It can be observed that the sequence is better conserved in the transmembrane regions, whereas the non-transmembrane region, especially the turn structure, is less conserved. In addition, we found that TMHs 5, 6, 7, and 8 were more conserved than TMHs 1, 2, 3, and 4.

### Construction of Recombinant Plasmid

**Figure 3:**
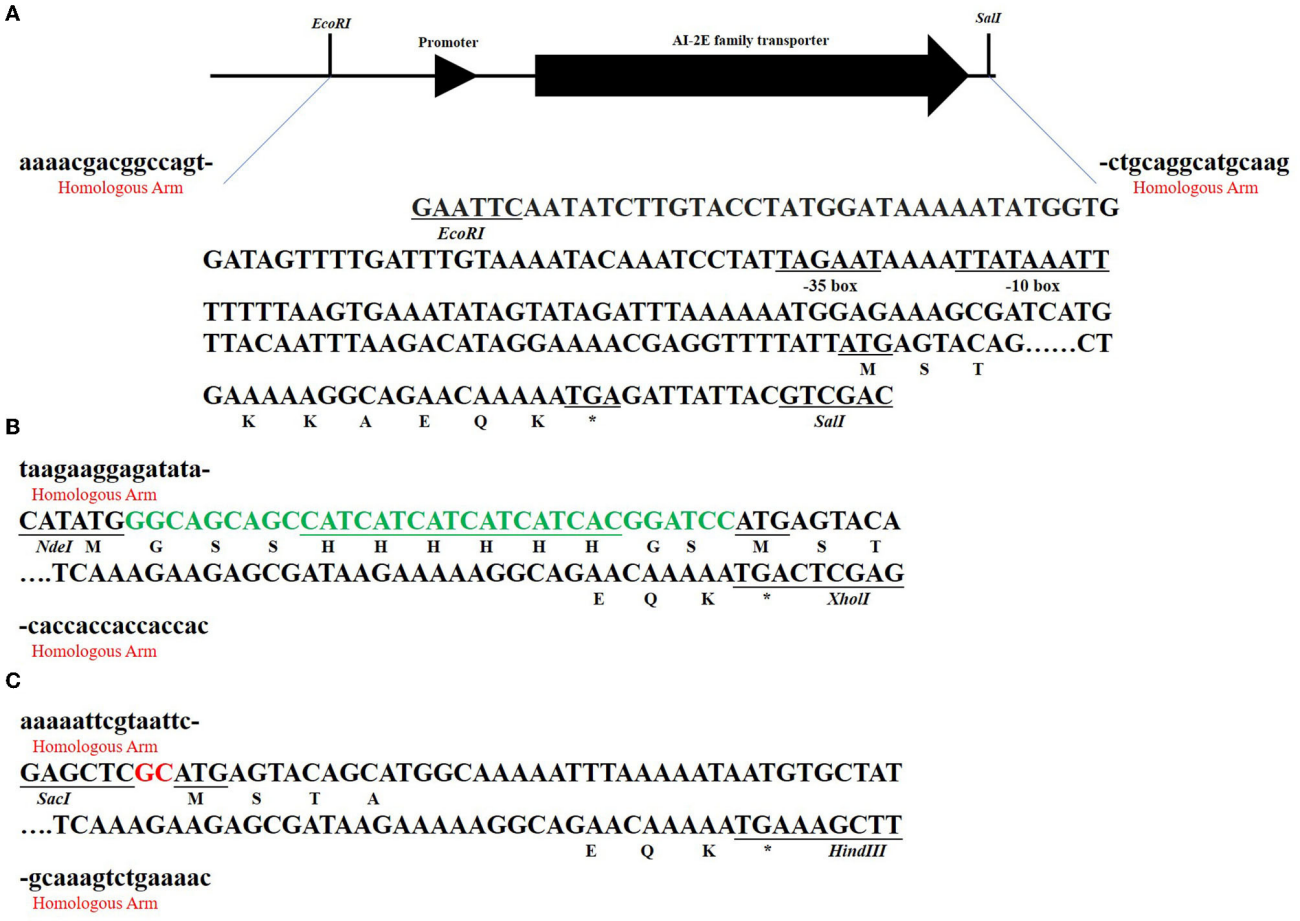
The subcloning strategy of the *AI-2E* gene. The sequences marked with red “homologous arm” represent the homologous arm when ligated to the vector, and the marked restriction endonucleases represent the two enzymes used in linearizing the vector. The asterisks represent the termination codon. The subcloning strategy of *AI-2E* gene and its promoter DNA fragment that for ligation to pUC19 vector **(A)**. The subcloning strategy of *AI-2E* gene that for ligation to pET22b vector **(B)**. Subcloning strategy for the AI-2E gene used to ligate to the pMG36e vector to construct an overexpression vector **(C)**.

Figure 3 shows the sketch of the framework of the three recombinant plasmids we constructed. As can be seen in Figure 3A, we predicted the promoter sequence of AI-2E and ligated it into the pUC19 plasmid after complete amplification of the entire fragment of interest. In Figure 3B, we completely amplified the coding sequence (CDS) of AI-2E and ligated it into the multiple cloning sites of pET22b, which we replaced with a 6 × His tag as the pelB tag of pET22b vector. To overexpress AI-2E protein in *L. acidophilus*, we fully amplified the CDS of AI-2E into the pMG36e expression vector, and a slight adjustment was made to ensure the accuracy of the transcription process (Figure 3C). The sequences of primers used in this study are shown in Table 1.

**Table 1:** Primers used in this study.

**Purpose of primer**	**Forward primer (5^**′**^-3^**′**^)**	**Reverse primer (5^**′**^-3^**′**^)**
Ligation to pUC19	AAAACGACGGCCAGTGAATTCAATATCTTGTACCTATGGATA AAAATATGGT	CTTGCATGCCTGCAGGTCGACGTAATAATCTCATTTTTGTT CTGCCTTT
Ligation to pET22b1	GGCAGCAGCCATCATCATCATCATCACGGATCCATGAGTACAGCA TGGCAAAAATTT	TCATTTTTGTTCTGCCTTTTTC
Ligation to pET22b2	TAAGAAGGAGATATACATATGGGCAGCAGCCATCATCATCA	GTGGTGGTGGTGGTGCTCGAGTCATTTTTGTTCTGCCTTTTTCTTA
Ligation to pMG36e	AAAAATTCGTAATTCGAGCTCGCATGAGTACAGCATGGCAAAAATTT	GTTTTCAGACTTTGCAAGCTTTCATTTTTGTTCTGCCTTTTTCTTA
MCS of pMG36e	CAATCTGCCTCCTCATCCT	CTGATCTCAACAATGTGAAGTC

### Salinity Tolerance Test of *E. coli* Strains

**Figure 4:**
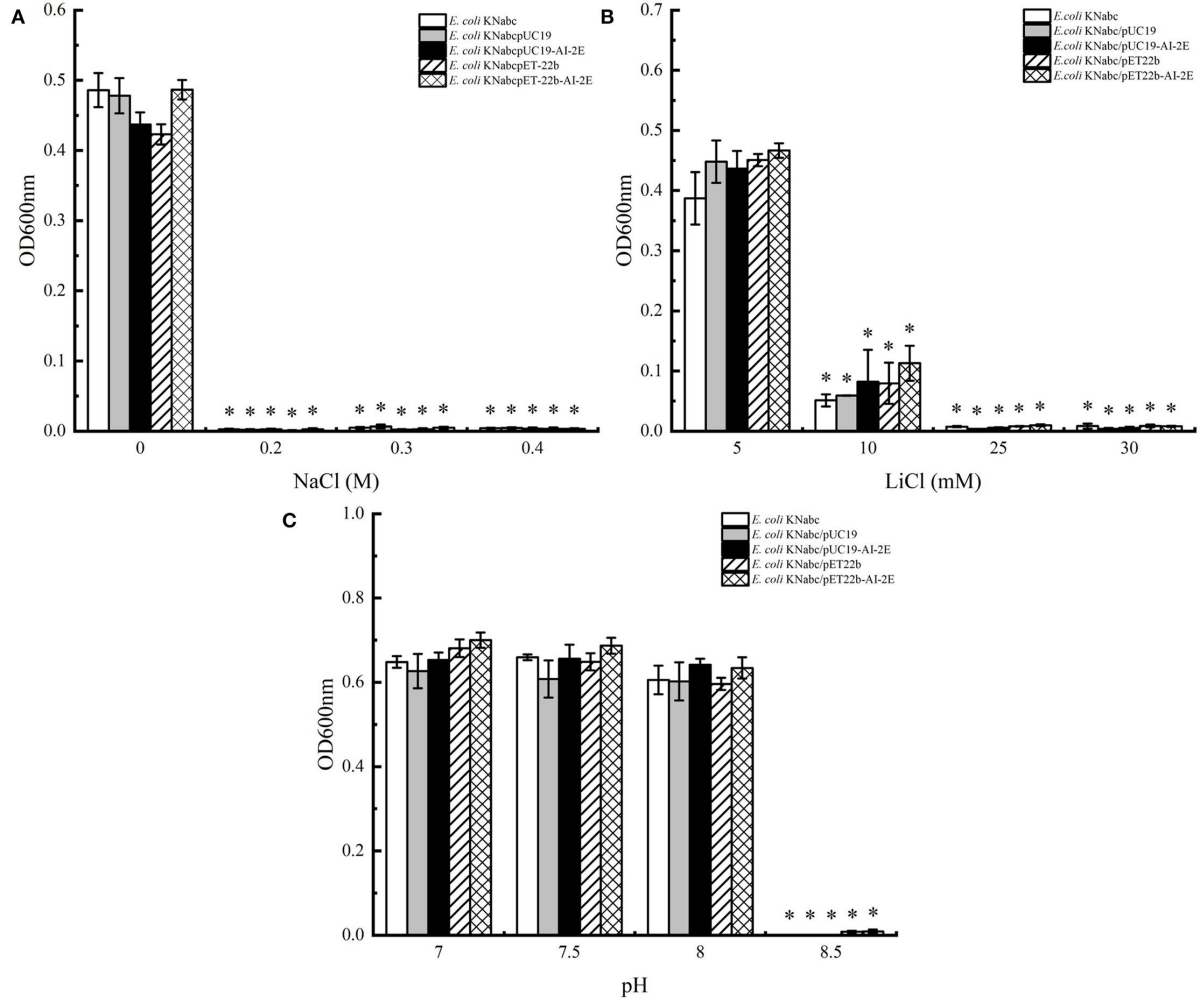
Salinity tolerance of *Escherichia coli* KNabc strains. Growth tests were carried out in the LBK media containing 0.2, 0.3, and 0.4 M NaCl **(A)**, 5, 10, 25, and 30 mM LiCl **(B)**, or at pH 7.0, 7.5, 8.0, and 8.5 **(C)**. Data are presented as mean ± SEM; *n* ≥ 3; *p < 0.05 compared with the wild-type strain.

The assay results of the resistance of *E. coli* KNabc wild-type strain and recombinant strains (*E. coli* KNabc/pUC19, *E. coli* KNabc/pUC19, *E. coli* KNabc/pET22b, and *E. coli* KNabc/pET22b-AI-2E) to salts and alkaline pH are shown in Figure 4. In the fresh LBK medium, all strains grew normally and were no significant difference in the OD_600_ values of all strains measured after 24 h, whereas the growth of all strains was inhibited by the addition of 0.2, 0.3, and 0.4 M NaCl to the LBK medium (Figure 4A). After adding 5 mM of LiCl to the fresh LBK medium, all strains grew normally or with low inhibition; all strains could grow slightly after adding 10 mM of LiCl and were inhibited completely after adding 25 and 30 mM of LiCl (Figure 4B). Figure 4C shows the growth of different strains in the LBK medium at different pH. We can see that all strains grew normally or were only very weakly inhibited in pH 7.0, 7.5, and 8.0, whereas the growth of all strains was inhibited completely in the LBK medium at pH 8.5. These phenomena suggest that the AI-2E protein of *L. acidophilus* does not recover the growth of the *E. coli* KNabc strains in saline environments. With the results we have observed so far, the AI-2E protein may not possess Na^+^ (Li^+^)/H^+^ antiporter activity.

### Overexpression of AI-2E Protein Promotes AI-2 Secretion in *L. acidophilus* CICC 6074

**Figure 5:**
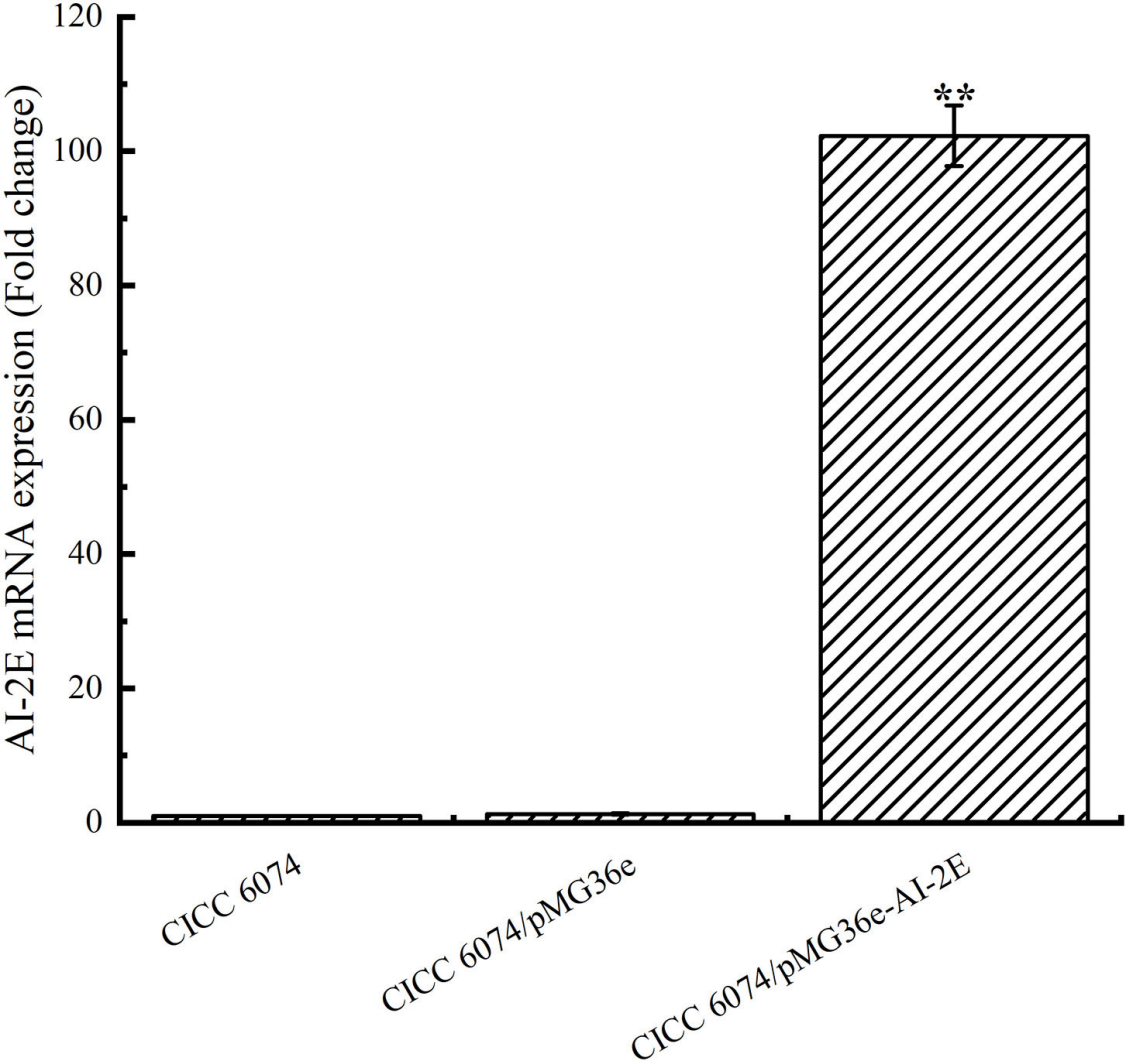
*AI-2E* gene mRNA expressions of *Lactobacillus acidophilus* CICC 6074 wild-type strain, *L. acidophilus* CICC 6074/pMG36e recombinant strain, and *L. acidophilus* CICC 6074/pmG36e-AI-2E recombinant strain. Data are presented as mean ± SEM; *n* ≥ 3; **p < 0.01 compared with the wild-type strain.

To verify whether the AI-2E gene was overexpressed in *L. acidophilus* CICC 6074 stain, RT-qPCR was used to detect the relative mRNA content of the AI-2E gene among different strains. As shown in Figure 5, the expression of the *AI-2E* gene was upregulated 102.29 times in the *L. acidophilus* CICC 6074/pMG36e-AI-2E relative to the wild-type strain, and no significant difference between the *L. acidophilus* CICC 6074/pMG36e strain and the wild-type strain was found.

### Detection of AI-2

**Figure 6:**
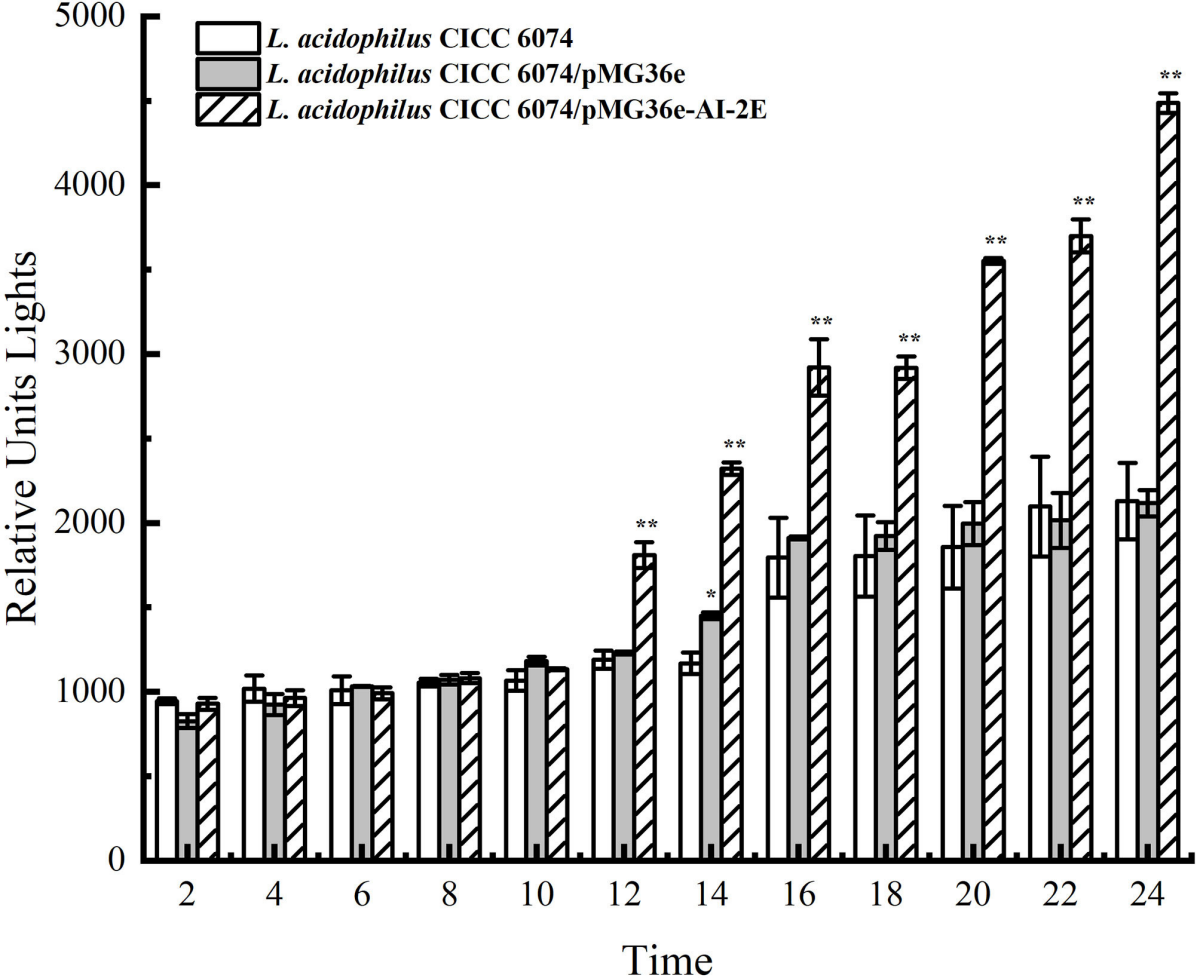
AI-2 activity in culture supernatant with the bacterial cells removed of *Lactobacillus acidophilus* CICC 6074 wild-type strain, *L. acidophilus* CICC 6074/pMG36e recombinant strain, and *L. acidophilus* CICC 6074/pmG36e-AI-2E recombinant strain. Data are presented as mean ± SEM; *n* ≥ 3; *p < 0.05, **p < 0.01 compared with the wild-type strain.

The AI-2E proteins are claimed to have the AI-2 exporter activity. To observe the effect of AI-2E overexpression on AI-2 secretion, we detected AI-2 activities in *L. acidophilus* CICC 6074 wild type strain and recombinant strains (*L. acidophilus* CICC 6074/pMG36e and *L. acidophilus* CICC 6074/pMG36e-AI-2E) over 24 h. As shown in Figure 6, AI-2 secretion by all strains increased slowly and did not differ significantly among strains in the first 10 h. *L. acidophilus* CICC 6074/pMG36e-AI-2E strain shows a rapid increase in AI-2 secretion between 12 and 24 h, which is significantly different from the other strains.

### Overexpression of AI-2E Protein Improved Intestinal Juice Resistances of *L. acidophilus* CICC 6074

**Figure 7:**
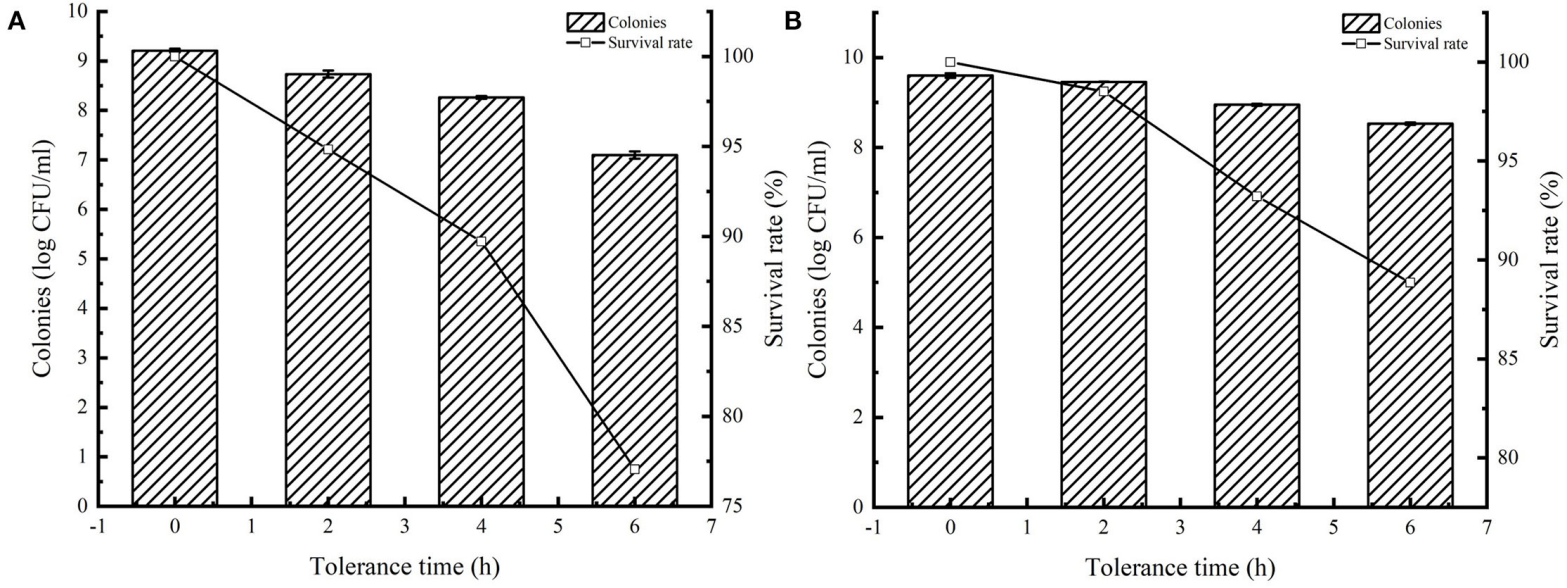
Tolerance of *Lactobacillus acidophilus* CICC 6074 strains in artificial intestinal juice. Survival of *L. acidophilus* CICC 6074/pMG36e recombinant strain after tolerance in artificial intestinal juice **(A)**. Survival of *L. acidophilus* CICC 6074/pMG36e-AI-2E recombinant strain after tolerance in artificial intestinal juice **(B)**.

Many studies have reported that AI-2 facilitates bacterial tolerance to the environment. Overexpression of AI-2E enhances AI-2 secretion, and here we tested the tolerance of AI-2E protein overexpressing strain in simulated intestinal juice, and the results are shown in Figure 7. Figure 7A shows the survival status of the recombinant strain *L. acidophilus* CICC 6074 in simulated intestinal juice tolerated for 6 h. We can see that the viable count of *L. acidophilus* CICC 6074/pMG36e strain decreased from 9.21 log CFU/ml to 7.09 log CFU/ml, and the survival rate decreased to 77.06%. And Figure 7B shows that the viable count of AI-2E overexpression strain decreased from 9.42 to 8.89 log CFU/ml after 6 h of intestinal fluid tolerance, and the survival rate decreased to 94.35%. These results indicate that the overexpression of AI-2E protein can effectively enhance the viability of the *L. acidophilus* CICC 6074 strain in the intestinal juice.

## Discussion

In the study of Wang et al. (2020) and Shao et al. (2021), the AI-2E protein from the *H. andaensis* and their homolog proteins were also assigned to a single branch during phylogenetic analysis. The function of proteins in this branch was identified as the Na^+^ (Li^+^)/H^+^ antiporter activity and is related to the salinity tolerance of microorganisms. During the phylogenetic analysis, we found that 530 AI-2E proteins belonging to *Lactobacillus* were divided into 11 large clades and some small clades, indicating that the AI-2E family transporter proteins of different species are not conserved. Proteins from different sources may have different functions, which is consistent with the findings of Wang et al. (2020). It was also found that proteins on the same branch tend to belong to several same species, which means that these AI-2E proteins may be relatively conserved within species. When the AI-2E protein of *Lactobacillus* and download from TCBD were used for phylogenetic analysis, it was found that most of the AI-2E proteins of *Lactobacillus* were allocated to one branch alone. The closest to this branch is AI-2 exporter group I. Interestingly, 6 other AI-2E proteins from *Lactobacillus* were assigned to AI-2 exporter group I. These suggest that the function of the AI-2E protein from *L*. *acidophilus* is most likely AI-2 exporter activity. In addition to protein amino acid sequence, protein structure is also a key point to consider when analyzing protein homology. We predicted the secondary structure and functional domains of AI-2E protein in *L*. *acidophilus* CICC 6074 strain cells, and the results show there are 8 transmembrane regions on the protein. In the study of Dong et al. (2017), 6 transmembrane domains were predicted in the UPF0118 protein with Na^+^ (Li^+^)/H^+^ antiporter activity. Herzberg et al. (2006) reported that it predicted 8 transmembrane regions from the ydgG protein with AI-2 exporter activity in the *E*. *coli* K-12 strain, which indicates that the AI-2E protein of *L. acidophilus* is closer to the protein with AI-2 secretion function at least in terms of the number of transmembrane regions.

To verify the function of AI-2E protein in the *L. acidophilus* CICC6074 strain, we expressed it in the *E. coli* KNabc strain and overexpressed it in the *L. acidophilus* CICC6074 strain. The results show that the expressed AI-2E protein could not improve the salt-alkali resistance of *E. coli* KNabc. But could increase the secretion of AI-2 by overexpressing the AI-2E protein in the *L. acidophilus* CICC 6074 strain. All results indicate that the AI-2E in *L. acidophilus* CICC 6074 strain cells may have AI-2 exporter activity. LAB use quorum sensing (QS) to communicate with each other. AI-2 is a signaling molecule for intra-species and inter-species communication. QS is closely related to the environmental adaptability, biofilm formation, and intestinal epithelial cell adhesion of LAB. The persistence of mutant strains in the gastrointestinal tract of mice was significantly reduced after the *LuxS* gene of *L. rhamnosus* GG was knocked out, and the resistance to gastric juice was recovered after gene backfilling (Lebeer et al., 2008). Jia et al. reported that LuxS/AI-2 is involved in the tolerance of LAB to the gastrointestinal environment. The survival rate of the *L. plantarum* KLDS1.0391 mutant strain with *LuxS* gene knockout in gastric acid and intestinal fluid was significantly lower than that of the wild-type strain (Jia et al., 2018).

Both Na^+^ (Li^+^)/H^+^ antiporter activity and AI-2 exporter activity are related to the environmental adaptability of microorganisms. Dong et al. (2017) found that the AI-2E protein with Na+ (Li+)/H+ antiporter activity is important for the moderate halophile *H. andaensis* that to survive in saline environments. They also found that the expression of AI-2E protein in the *E. coli* KNabc strain could restore the resistance of this strain to salinity (Dong et al., 2017). Buck et al. (2009) reported that AI-2 could enhance the resistance of *L. acidophilus* NCFM to low pH and bile salts. Liu et al. (2018) demonstrated that overexpression of the LuxS protein could produce more AI-2, it could enhance the resistance of *L*. *paraplantarum* L-ZS9 strain to heat stress and bile salts. These studies indicate that AI-2 can improve the adaptability of LAB to these environments. In bad conditions, LAB can enhance the activity of the AI-2 by increasing the transcription level of the *LuxS* gene, and the presence of the AI-2/LuxS QS system in bacteria can help bacteria survive in the gastrointestinal tract. In our study, we overexpressed the AI-2E protein in the *L. acidophilus* CICC 6074 strain, and the AI-2 activity of this strain has also significantly increased. The results of intestinal juice tolerance show that more AI-2 can significantly improve the survival rate of *L. acidophilus* cells in intestinal juice.

## Conclusion

In our study, we found that expressing the AI-2E protein in the *E. coli* KNabc strain did not recover the resistance of the bacteria to the saline environment, indicating that the AI-2E protein may not possess Na^+^ (Li^+^)/H^+^ antiporter activity. Overexpression of AI-2E protein in *L. acidophilus* CICC 6074 strain resulted in more AI-2 secretion by the cells, which proves that the AI-2E protein of *L. acidophilus* may have the AI-2 exporter activity. The survival rate of the AI-2E protein overexpression strain is significantly higher than the wild-type strain when growing in intestinal juice. These results indicate that overexpression of AI-2E protein in *L. acidophilus* CICC 6074 strain could promote to secrete of more AI-2, thereby enhancing the viability of LAB cells in intestinal juice.

## Data Availability Statement

The original contributions presented in the study are included in the article/supplementary material, further inquiries can be directed to the corresponding author/s.

## Author Contributions

XL, ZS, and DP conceived and designed the experiments. XL, JX, and YC performed the experiments. XL, TZ, and XF performed bioinformatics analysis. XL wrote the manuscript. DP funded all the expenses for this article. All authors checked and approved the final version of this manuscript.

## Funding

This work was supported by the Natural Science Foundation of China (31972048), the Science Technology Department of Ningbo (2019C10017 and 202002N3076), and the National Key R&D Program of China (2021YFD2100104).

## Conflict of Interest

The authors declare that the research was conducted in the absence of any commercial or financial relationships that could be construed as a potential conflict of interest.

## Publisher's Note

All claims expressed in this article are solely those of the authors and do not necessarily represent those of their affiliated organizations, or those of the publisher, the editors and the reviewers. Any product that may be evaluated in this article, or claim that may be made by its manufacturer, is not guaranteed or endorsed by the publisher.
